# Trinucleotide CGG Repeat Diseases: An Expanding Field of Polyglycine Proteins?

**DOI:** 10.3389/fgene.2022.843014

**Published:** 2022-02-28

**Authors:** Manon Boivin, Nicolas Charlet-Berguerand

**Affiliations:** Institut de Génétique et de Biologie Moléculaire et Cellulaire (IGBMC), INSERM U 1258, CNRS UMR 7104, University of Strasbourg, Illkirch, France

**Keywords:** microsatellite, protein aggregates toxicity, RAN translation, neurodegeneration, trinucleotide repeat disorders

## Abstract

Microsatellites are repeated DNA sequences of 3–6 nucleotides highly variable in length and sequence and that have important roles in genomes regulation and evolution. However, expansion of a subset of these microsatellites over a threshold size is responsible of more than 50 human genetic diseases. Interestingly, some of these disorders are caused by expansions of similar sequences, sizes and localizations and present striking similarities in clinical manifestations and histopathological features, which suggest a common mechanism of disease. Notably, five identical CGG repeat expansions, but located in different genes, are the causes of fragile X-associated tremor/ataxia syndrome (FXTAS), neuronal intranuclear inclusion disease (NIID), oculopharyngodistal myopathy type 1 to 3 (OPDM1-3) and oculopharyngeal myopathy with leukoencephalopathy (OPML), which are neuromuscular and neurodegenerative syndromes with overlapping symptoms and similar histopathological features, notably the presence of characteristic eosinophilic ubiquitin-positive intranuclear inclusions. In this review we summarize recent finding in neuronal intranuclear inclusion disease and FXTAS, where the causing CGG expansions were found to be embedded within small upstream ORFs (uORFs), resulting in their translation into novel proteins containing a stretch of polyglycine (polyG). Importantly, expression of these polyG proteins is toxic in animal models and is sufficient to reproduce the formation of ubiquitin-positive intranuclear inclusions. These data suggest the existence of a novel class of human genetic pathology, the polyG diseases, and question whether a similar mechanism may exist in other diseases, notably in OPDM and OPML.

## Introduction

DNA short tandem repeats (STR), also known as microsatellites, are short sequences of 3–6 nucleotides repeated multiple time so that they occupy as much as 2–3% of the human genome. These microsatellites are highly variable in size and sequence and have important roles in genomes regulation and evolution. However, expansion of a subset of these microsatellite sequences over a threshold size can also be the leading cause of human genetic diseases, and 2021 marks the 30th anniversary of the discovery of the two first pathogenic trinucleotide expansions, namely CGG and CAG repeats located in the fragile X mental retardation (FMR1) and androgen receptor (AR) genes and that cause fragile X syndrome (FXS) and spinal and bulbar muscular atrophy (SBMA), respectively (Fu et al., 1991; Kremer et al., 1991; La Spada et al., 1991; Oberlé et al., 1991; Verkerk et al., 1991). Since then, expansion of tri-, tetra-, penta- and hexa-nucleotide sequences were identified in more than 50 neurodevelopmental, neuromuscular and neurodegenerative genetic disorders (review in Malik et al., 2021a; Depienne and Mandel, 2021; Table 1). These microsatellite mutations are highly variable with expansion lengths changing among populations, generation and individuals, and even according to tissues, cells and/or age. Thus, in a subgroup of microsatellite diseases where clinical manifestations correlate with expansion sizes, symptoms may greatly diverge between individuals and/or between generations, resulting in atypical heritability such as anticipation (increased disease severity and/or decreased age of onset with increased repeat size among generations) or its opposite mechanism, contraction where the expansion may shrink below its pathogenic threshold size from one generation to another. Moreover, these repeat expansions can present a profound genetic founder effect, restricting observation of some of these mutations to specific human populations. For example, the G4C2 repeat expansion in the C9ORF72 gene, which causes amyotrophic lateral sclerosis (ALS) and frontotemporal dementia (FTD), is amply observed in North Europe and North America but rare in other populations; while a CGG repeat expansion in the NOTCH2NLC gene that causes neuronal intranuclear inclusion disease (NIID) is increasingly reported in Asian populations, but rare in Europe. Finally, these microsatellite expansions can be located in either non-transcribed and non-coding sequences, transcribed but untranslated regions, or within translated sequences, and 30 years of researches have now unveiled that these expansions are pathogenic by both loss and/or gain of function mechanisms at the DNA, RNA, and protein levels (review in Malik et al., 2021a; Depienne and Mandel, 2021; Table 1).

**TABLE 1:** Repeat expansion diseases, sorted by their proposed pathogenic mechanism.

Proposed mechanism	Disease	Gene	Localization	Repeat	Normal	Pathogenic	Reference
					size	size	
LOF	BSS	*XYLT1*	Promoter	CGG	9–20	120–800	LaCroix et al. (2019)
LOF	FXS	*FMR1*	5′ UTR	CGG	5–50	>200	Verkerk et al. (1991); Oberlé et al. (1991); Fu et al. (1991)
LOF	FRAXE	*AFF2*	5′ UTR	CCG	4–39	200–900	Knight et al. (1993)
LOF	EPM1	*CSTB*	5′ UTR	C4GC4GCG	2–3	30–75	Lalioti et al., 1997
LOF	GDPAG	*GLS*	5′ UTR	GCA	8–16	680–1400	Van Kuilenburg et al. (2019)
LOF	FRDA	*FXN*	Intron	GAA	5–34	65–1300	Campuzano et al. (1996)
LOF	XDP	*TAF1*	Intron	C3TCT	absent	30–55	Bragg et al. (2017)
polyAla	SPD1	*HOXD13*	Exon	GCG	15	24	Akarsu et al. (1996)
polyAla	BCCD	*RUNX2*	Exon	GCN	17	27	Mundlos et al. (1997)
polyAla	HFGS	*HOXA13*	Exon	GCN	12–18	18–30	Goodman et al. (2000)
polyAla	BPES	*FOXL2*	Exon	GCN	14	19–24	De Baere et al. (2001)
polyAla	HPE5	*ZIC2*	Exon	GCN	15	25	Brown et al. (2001)
polyAla	EIEE1	*ARX*	Exon	GCN	12–16	20–23	Stromme et al. (2002)
polyAla	MRGH	*SOX3*	Exon	GCN	15	26	Laumonnier et al. (2002)
polyAla	CCHS	*PHOX2B*	Exon	GCN	20	25–29	Amiel et al. (2003)
polyAla	OPMD	*PABPN1*	Exon	GCG	6–10	11–18	Brais et al. (1998)
polyQ	SBMA	*AR*	Exon	CAG	9–36	38–68	La Spada et al. (1991)
polyQ	DRPLA	*ATN1*	Exon	CAG	3–35	48–93	Koide et al. (1994); Nagafuchi et al. (1994)
polyQ	HD	*HTT*	Exon	CAG	6–35	36–200	Huntington’s Collaborative Group (1993)
polyQ	HDL2	*JPH3 AS*	Exon	CAG	6–28	41–58	Margolis et al. (2001)
polyQ	SCA1	*ATXN1*	Exon	CAG	6–38	39–88	Orr et al. (1993)
polyQ	SCA2	*ATXN2*	Exon	CAG	13–31	32–500	Pulst et al. (1996)
polyQ	SCA3	*ATXN3*	Exon	CAG	12–44	55–87	Kawaguchi et al. (1994)
polyQ	SCA6	*CACNA1A*	Exon	CAG	4–18	20–33	Zhuchenko et al. (1997)
polyQ	SCA7	*ATXN7*	Exon	CAG	4–33	37–460	Lindblad et al. (1996)
polyQ	SCA8	*ATXN8*	Exon	CAG	15–50	74–250	Koob et al. (1999)
polyQ	SCA17	*TBP*	Exon	CAG	25–40	43–66	Koide et al. (1999)
?	SCA12	*PPP2R2B*	5′ UTR	CAG	4–32	43–78	Holmes et al. (1999)
polyGly	FXTAS	*FMR1*	5′ UTR	CGG	5–50	55–200	Hagerman et al. (2001)
polyGly	NIID	*NOTCH2NLC*	5′ UTR	CGG	7–60	60–200	Ishiura et al. (2019); Sone et al. (2019); Tian et al. (2019); Deng et al. (2019)
?	FXPOI	*FMR1*	5′ UTR	CGG	5–50	55–200	Conway et al. (1998)
?	OPML	*LOC642361*	LncRNA	CGG	3–16	50–60	Ishiura et al. (2019)
?	OPDM1	*LRP12*	5′ UTR	CGG	13–45	80–130	Ishiura et al. (2019)
?	OPDM2	*GIPC1*	5′ UTR	CGG	12–32	70–120	Deng et al. (2020)
?	OPDM3	*NOTCH2NLC*	5′ UTR	CGG	7–60	60–200	Yu et al. (2021)
RAN	ALS/FTD	*C9ORF72*	Intron	G4C2	3–25	>30	Dejesus-Hernandez et al. (2011); Renton et al. (2011)
RAN	SCA36	*NOP56*	Intron	G3C2T	5–14	650–2,500	Kobayashi et al. (2011)
RAN	SCA31	*BEAN1*	Intron	G2A2T	variable	110–760	Sato et al. (2009)
?	CANVAS	*RFC1*	Intron	G3A2	variable	400–2000	Cortese et al. (2019); Rafehi et al. (2019)
RNA	DM1	*DMPK*	3′ UTR	CTG	5–37	50–10,000	Mahadevan et al. (1992); Brook et al. (1992); Fu et al. (1992)
RNA	DM2	*CNBP*	Intron	CCTG	11–30	50–11,000	Liquori et al. (2001)
RNA	FECD3	*TCF4*	Intron	CTG	5–31	>50	Mootha et al. (2014)
?	FAME1	*SAMD12*	Intron	TTTCA	absent	440–3,680	Ishiura et al. (2018)
?	FAME2	*STARD7*	Intron	TTTCA	absent	>660–730	Corbett et al. (2019)
?	FAME3	*MARCHF6*	Intron	TTTCA	absent	>660–2,800	Florian et al. (2019)
?	FAME4	*YEATS2*	Intron	TTTCA	absent	>500	Yeetong et al. (2019)
?	FAME6	*TNRC6A*	Intron	TTTCA	absent	>400	Ishiura et al. (2018)
?	FAME7	*RAPGEF2*	Intron	TTTCA	absent	>500	Ishiura et al. (2018)
?	SCA10	*ATXN10*	Intron	TTCTA	10–32	280–4,500	Matsuura et al. (2000)
?	SCA37	*DAB1*	Intron	TTTCA	absent	31–75	Seixas et al. (2017)

LOF, loss of function mechanism; polyAla, polyalanine; polyGly, polyglycine; polyQ, polyglutamine; RAN, repeat non-ATG, translation; ALS, amyotrophic lateral sclerosis; BCCD, brachydactyly and cleidocranial dysplasia; BPES, blepharophimosis, ptosis and epicanthus inversus; BSS, Baratela-Scott syndrome; CANVAS, cerebellar ataxia, neuropathy and vestibular areflexia syndrome; CCHS, congenital central hypoventilation syndrome; DM1, myotonic dystrophy type 1; DM2, myotonic dystrophy type 2; DRPLA, dentatorubral-pallidoluysian atrophy; EIEE1, early infantile epileptic encephalopathy type 1; EPM1, progressive myoclonus epilepsy type 1; FAME, familial adult myoclonic epilepsy; FECD3, Fuchs endothelial corneal dystrophy type 3; FRAXE, fragile XE, syndrome; FRDA, Friedreich ataxia; FTD, frontotemporal dementia/; FXPOI, Fragile X-associated premature ovarian infertility; FXS, fragile X syndrome; FXTAS, fragile X-associated tremor ataxia syndrome; GDPAG, global developmental delay, progressive ataxia and elevated glutamine; HD, Huntington disease; HDL2, Huntington disease-like 2; HFGS, hand-foot-genital syndrome; HPE5, holoprosencephaly type 5; MRGH, mental retardation with isolated growth hormone deficiency; NIID, neuronal intranuclear inclusion disease; OPDM, oculopharyngodistal myopathy type; OPMD, oculopharyngeal muscular dystrophy; OPML, oculopharyngeal myopathy with leukoencephalopathy; SBMA, spinal and bulbar muscular atrophy; SPD1, synpolydactyly type 1; SCA, spinocerebellar ataxia; XDP, X-linked dystonia parkinsonism.

First, repeat expansions located within or close to promoters can promote DNA epigenetic changes that inhibit transcription, resulting in reduced expression of the encoded protein. As an indication that these expansions are pathogenic through a loss of function mechanism, identical clinical manifestations can be observed in individuals carrying genetic deletions and/or loss of function coding mutations of the gene hosting these microsatellites. Archetype of expansions shutting down gene expression are the GGC, CGG, and GAA expanded repeats located within the promoter, 5′UTR and first intron of the XYLT1, FMR1, and frataxin genes and associated with Baratela-Scott syndrome (BBS), fragile X syndrome (FXS), and Friedreich ataxia (FA), respectively, (Fu et al., 1991; Oberlé et al., 1991; Verkerk et al., 1991; Kremer et al., 1991; Campuzano et al., 1996; laCroix et al., 2019; Table 1).

Second, these expansions can be pathogenic at the RNA level through a toxic RNA gain of function mechanism. In these disorders, large repeat expansions are transcribed into pathogenic RNA that accumulate into nuclear RNA foci, which recruit and consequently alter the localization and function of specific RNA binding proteins, ultimately resulting in multiple RNA processing defects that are responsible of the diverse clinical manifestations observed in these diseases (review in Wheeler and Thornton, 2007; Morriss and Cooper, 2017; Sznajder and Swanson, 2019). This RNA gain of function mechanism is best exemplified by the titration of the Muscleblind-like 1 and 2 (MBNL) RNA binding proteins into nuclear RNA foci constituted of CUG or CCUG RNA repeat expansions in myotonic dystrophy type 1 and 2 (DM1 and 2), as well as in Fuchs endothelial corneal dystrophy (FECD) (Miller et al., 2000; Fardaei et al., 2002; Wieben et al., 2017; Rong et al., 2019). These three disorders are caused by similar mutations, namely large expansions of CTG, CCTG, and CTG repeats embedded within the 3′UTR of DMPK or within introns of the CNBP and TCF4 genes, respectively, (Brook et al., 1992; Fu et al., 1992; Harley et al., 1992; Mahadevan et al., 1992; Liquori et al., 2001; Wieben et al., 2012; Mootha et al., 2014; Table 1).

**FIGURE 1:**
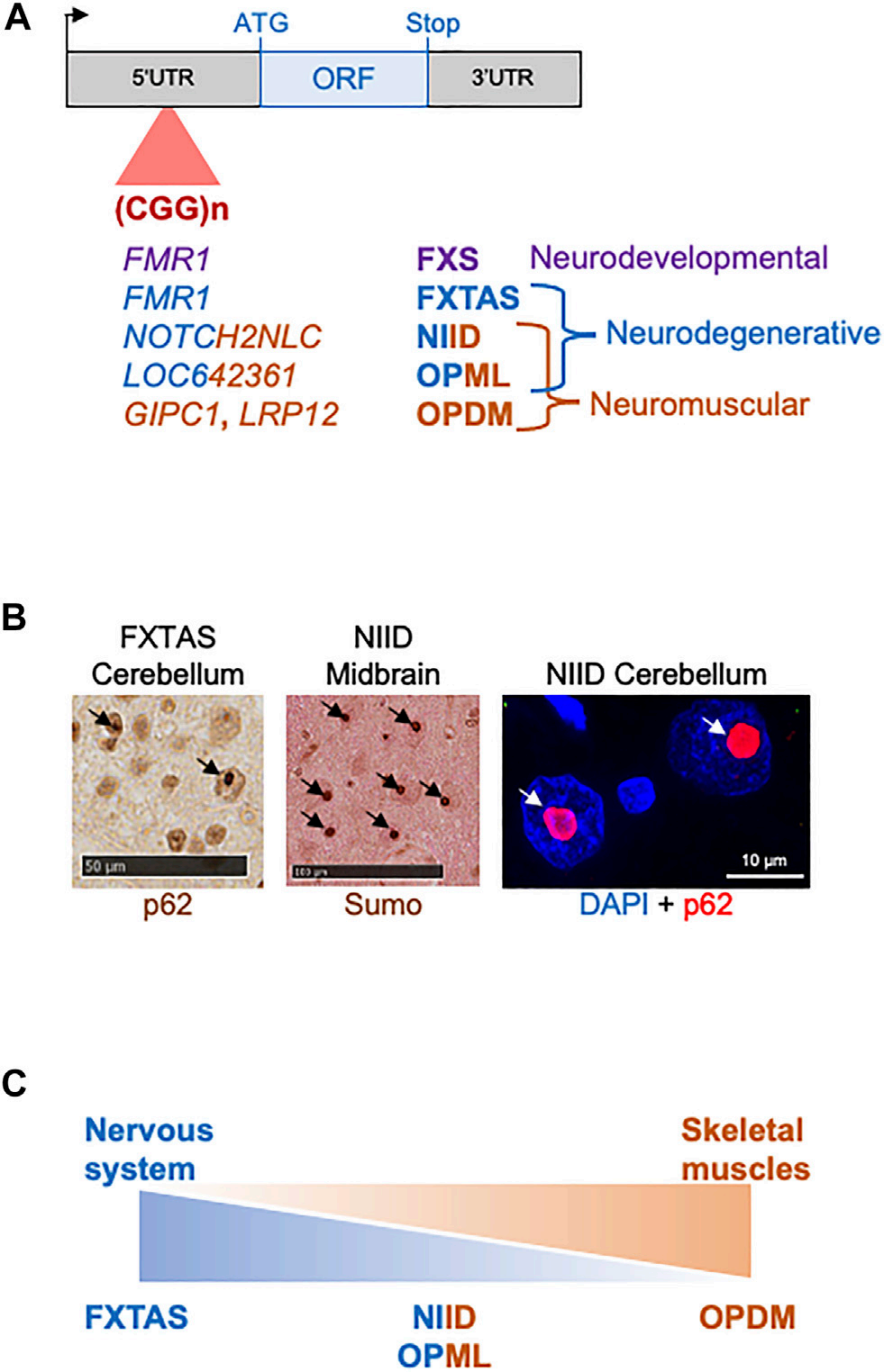
CGG repeat expansions cause a spectrum of disease. **(A)** Identical CGG repeat expansions embedded within the 5′UTR of different genes cause various neurodevelopmental, neuromuscular and neurodegenerative disorders. **(B)** Brain sections of individuals with FXTAS or NIID show identical p62-or sumo-positive intranuclear inclusions. **(C)** FXTAS, NIID, OPML, and OPDM may belong to a continuum of neuromuscular and neurodegenerative disorders.

Third, these repeat expansions can be translated into pathogenic proteins containing a stretch of repeated amino acids, resulting in a toxic gain of function, a loss of function, a dominant negative effect and/or a mix of these mechanisms for the protein hosting the expansion. Importantly, translation of these repeat expansions can occur through two main mechanisms. The first one is based on classical translation initiation at a canonical AUG, or alternatively at a near-cognate (CUG, GUG, UUG, or ACG), start codon, and thus results in expression of a pathogenic protein encoded by one predominant coding frame. The second mechanism, named repeat-associated non-AUG (RAN) translation, uses unconventional translation initiation that starts directly within the repeat expansion, resulting in expression of diverse proteins encoded in the three possible frames (Zu et al., 2011; review in Gao et al., 2017; Cleary et al., 2018; Nguyen et al., 2019). Archetype of this protein gain of function mechanism is the polyglutamine (polyQ) group of diseases, where expansions of CAG repeats, embedded within the main ORF of various genes (Huntingtin, Androgen receptor, Atrophin-1, ATXN1 to 3, 6, 7, and 17) (review in Lieberman et al., 2019), or alternatively within small ATG-driven ORFs located in ill-defined transcribed genetic regions (JPH3AS, ATXN8) (Moseley et al., 2006; Wilburn et al., 2011), are translated in polyglutamine-containing proteins that form inclusions and are toxic for neuronal cells, causing several neurodegenerative diseases with similar histopathological characteristic and related symptoms (Huntington’s disease and Huntington disease-like 2, spinal-bulbar muscular atrophy, dentatorubral-pallidoluysian atrophy, spinocerebellar ataxia 1–3, 6–8, and 17; Table 1). This example illustrates that repeat expansions with similar sequences, sizes, and genetic localizations can be pathogenic by a common molecular mechanism, ultimately resulting in a group of diseases with related clinical symptoms and remarkably similar histopathological features. In that aspect, progress in long-read and whole human genome sequencing has recently unveiled a dozen of novel pathogenic repeat expansions with striking similarities in sequence and localization and that can be divided in two groups (review in Ishiura and Tsuji, 2020). First, six identical expansions of ATTTC repeats that are all localized within introns, but of various gene, are responsible of familial adult myoclonic epilepsy (FAME) 1–4, 6, and 7 (Ishiura et al., 2018; Corbett et al., 2019; Florian et al., 2019; Yeetong et al., 2019). Second and topic of this review, several identical CGG repeat expansions, embedded within the 5′UTR of different genes or in a long non coding RNA (lncRNA), were recently identified as the causes of fragile X-associated tremor/ataxia syndrome (FXTAS), neuronal intranuclear inclusion disease (NIID), oculopharyngodistal myopathy type 1 to 3 (OPDM), and oculopharyngeal myopathy with leukoencephalopathy (OPML) (Hagerman et al., 2001; Ishiura et al., 2019; Sone et al., 2019; Deng et al., 2019; Tian et al., 2019; Deng et al., 2020; Xi et al., 2021; Table 1 and Figure 1). In addition to their similar genetic cause, FXTAS, NIID, OPDM, and OPML present overlapping clinical manifestations and similar histopathological features, including the presence of characteristic eosinophilic ubiquitin-positive nuclear inclusions (NIs), suggesting that these diseases belong to a continuum of neuromuscular and neurodegenerative diseases potentially caused by a common molecular mechanism (Figure 1).

## Fragile X-Associated Tremor/Ataxia Syndrome

Fragile X-associated tremor/ataxia syndrome (FXTAS, OMIM: 300623) is a late-onset X-linked neurodegenerative disorder originally described in grandfathers of infants with fragile X (Hagerman et al., 2001). Due to its X-linkage, FXTAS mostly affect males, while females are protected from overt neurodegeneration by random X-chromosome inactivation of the mutant allele, but are nonetheless at risk of developing another disease, fragile X-associated primary ovarian insufficiency (FXPOI; Conway et al., 1998). FXTAS is characterized by variable progressive intention tremor, gait ataxia and dementia, frequently accompanied by progressive cognitive decline, parkinsonism, peripheral neuropathy, and autonomic dysfunctions (Jacquemont et al., 2003). Neuroradiological features of FXTAS include mild brain atrophy and white matter lesions with T2 hyperintensities in the middle cerebellar peduncle, as well as in the splenium of the corpus callosum, in the pons, insula and periventricular white matter. Furthermore, FXTAS is characterized by the presence of large eosinophilic ubiquitin-, p62-, and sumo-positive intranuclear inclusions in both neurons and astrocytes across the nervous system, as well as in non-nervous tissues (Greco et al., 2006; Hunsaker et al., 2011; Ariza et al., 2016). However, these inclusions proved to be negative for polyQ or any other common protein inclusion markers of neurodegenerative disorders (Synuclein, Tau, ß-amyloid, TDP43, and FUS, etc.) and they origin remained mysterious until recently (Todd et al., 2013).

FXTAS is caused by a limited expansion (called premutation) of 55–200 CGG repeats localized in the 5′UTR of the *FMR1* gene (Hagerman et al., 2001). Interestingly, a longer expansion (>200 CGG repeats, named full mutation) causes the neurodevelopmental fragile X mental retardation syndrome, while typical individuals carry less than 45 CGG repeats, with 30 repeats being the most common allele. Importantly, the instable nature of this genetic mutation, notably repeat expansion during meiosis, explains the anticipation (also known as the Sherman paradox) observed in fragile X families where the clinical manifestations occur at progressively earlier age and/or with increasing severity in successive generations (Sherman et al., 1984; Sherman et al., 1985; Fu et al., 1991). At the molecular level, in fragile X syndrome, the full mutation promotes DNA epigenetic changes leading to transcriptional silencing of the *FMR1* promoter and thus, loss of expression of the *FMR1*-encoded protein, FMRP, ultimately resulting in autism and intellectual disability in male. In female, FMRP haploinsufficiency is less marked due to expression by the second unmethylated *FMR1* allele. In contrast, premutation expansions (55–200 CGG) enhance transcription, resulting in 2 to 8-fold increased levels of *FMR1* mRNA in individuals with FXTAS (Tassone et al., 2000; Kenneson et al., 2001; Tassone et al., 2007). In spite of this, FMRP expression are often decreased or at near-normal level due to the reduced translation efficiency of transcripts carrying elongated CGG repeats (Feng et al., 1995). Importantly, the sole expression of expanded CGG repeats, embedded in the 5′UTR of *FMR1*, in cell or animal models is sufficient to cause neuronal cell dysfunctions and to induce formation of the typical FXTAS intranuclear inclusions (Jin et al., 2003; Willemsen et al., 2003; Arocena et al., 2005; Entezam et al., 2007; Hashem et al., 2009; Todd et al., 2013; Hukema et al., 2015). These pioneering studies indicate that FXTAS, in contrary to the fragile X syndrome, is not due to a reduce expression of FMRP and to a loss of function mechanism, but is more likely caused by an RNA and/or protein gain of function mechanism.

About a toxic RNA gain of function mechanism in FXTAS*, in vitro* assays revealed that multiple RNA binding proteins bind to CGG RNA repeats, including Pura, hnRNP A2, SAM68, CUGBP1, TDP43, and Drosha/DGCR8, among others (Iwahashi et al., 2006; Jin et al., 2007; Sellier et al., 2010; Sofola et al., 2007; Sellier et al., 2013; Cid-Samper et al., 2018). However, whether CGG expansions are toxic at the RNA level in FXTAS is unclear. It notably remains to clarify whether these CGG RNA repeats deplete a sufficient amount of these RNA binding proteins, so that they loss their mobility and functions below a pathological threshold. As a further note of caution, many studies, including ours, were performed with CGG repeats deleted of any natural *FMR1* sequence and that consequently accumulate artificially in nuclei in some cell models, potentially titrating RNA binding proteins in nuclear RNA foci (Sellier et al., 2010). However, expanded CGG repeats embedded in their natural *FMR1* 5′UTR are exported into the cytoplasm and thus may not accumulate sufficiently into the nucleus to drive a nuclear RNA gain of function toxicity mechanism (Sellier et al., 2017). In that aspect, it remains to test whether exported CGG RNA repeats may accumulate into cytoplasmic RNA granules and titrate specific RNA binding proteins. Interestingly and notwithstanding their level of titration, these RNA binding proteins may also chaperone CGG RNA repeats to modulate their stability, localization and translation, resulting in diminished toxicity, thus illuminating a potential therapeutic strategy for FXTAS (Jin et al., 2007; Qurashi et al., 2011; He et al., 2014; Malik et al., 2021b).

**FIGURE 2:**
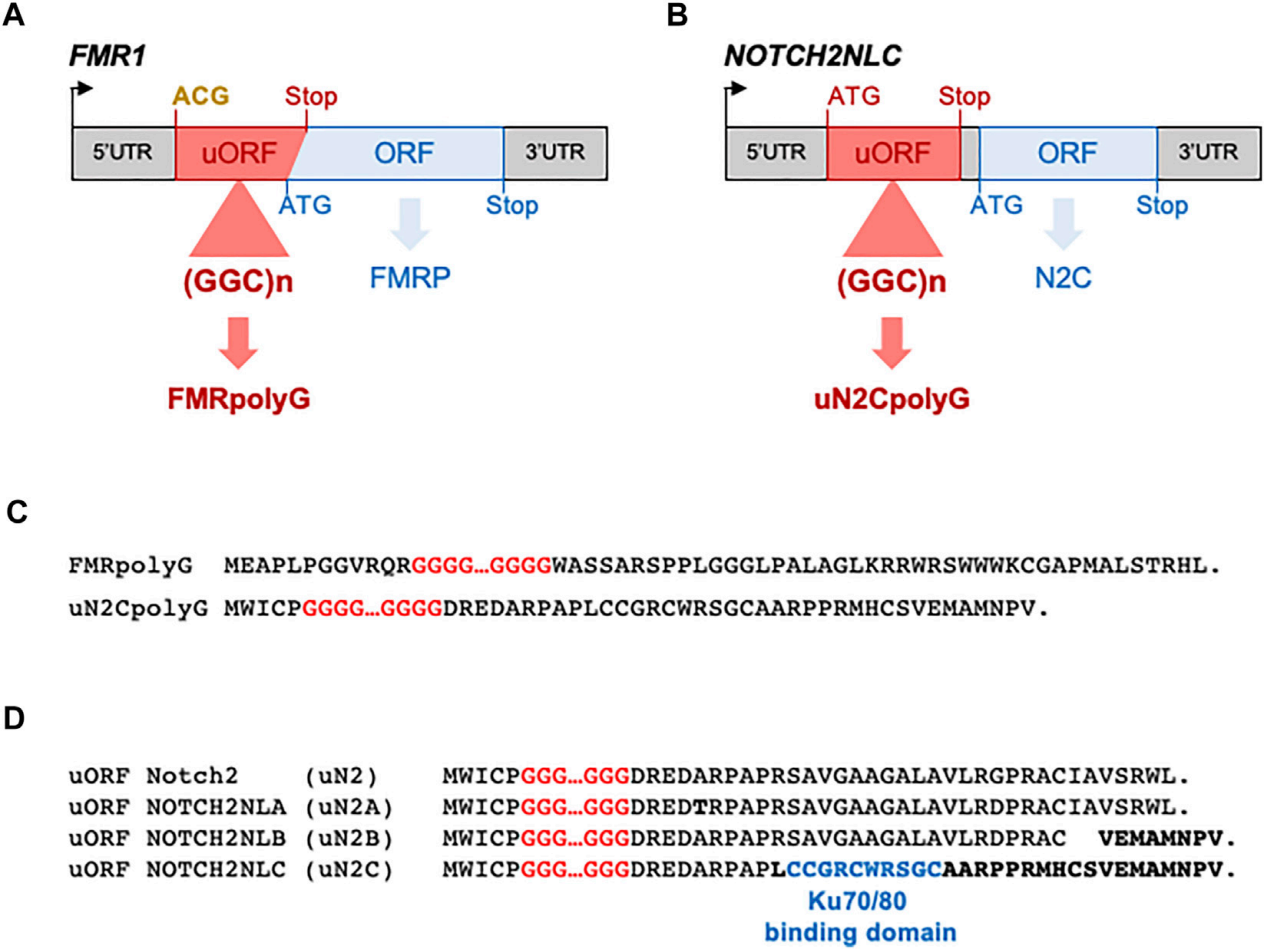
CGG repeat expansions are translated into polyG proteins in FXTAS and NIID. **(A)** Scheme of FMR1 indicating FMRpolyG upstream ORF and FMRP main ORF localization. **(B)** Scheme of NOTCH2NLC indicating uN2CpolyG upstream ORF and NOTCH2NLC (abbreviated N2C) main ORF localization. Initiation codons and stop codons are indicated in red for uORFs and in blue for the main ORFs. Near-cognate initiations codons are indicated in bold yellow. **(C)** Protein sequences of FMRpolyG and uN2CpolyG show no similitude beyond their polyglycine stretch. **(D)** Sequences of the putative uORFs embedded within NOTCH2, NOTCHNLA, B and C 5′UTRs. Variant amino acids are indicated in bold. The sequence required for uN2C to interact with KU70/KU80 is indicated in blue.

Concerning a pathogenic protein gain of function in FXTAS, studies of cell and animal models coupled to mass spectrometry analyses revealed that the *FMR1* CGG expansion is translated into a novel protein, where each GGC triplet encodes for a glycine, resulting in a small polyglycine-containing protein (polyG), which was named FMRpolyG (Todd et al., 2013). Of interest, translation of the *FMR1* CGG repeats occurs in one principal frame (glycine) among the three possible (alanine, glycine and arginine), but in absence of any ATG start codon. Instead, translation initiation takes place at an ACG near-cognate codon located 36 nucleotides upstream of the CGG repeats and in the glycine frame (Kearse et al., 2016; Sellier et al., 2017, Figure 2). Near-cognate initiation codons are codons differing from the cognate AUG start codon by one nucleotide, but that can still initiate translation through mispairing with the initiator methionine-tRNA. *In vitro* experiments and large-scale ribosome profiling revealed that predominantly four near-cognate start codons (CUG, GUG, UUG, and ACG) are tolerated and can initiate translation, despite fidelity control, notably by the eIF1 protein (Clements et al., 1988; Kozak, 1989; Peabody, 1989, Ingolia et al., 2011; Lee et al., 2012; review in Kearse and wilusz, 2017). However, translation initiation at near-cognate codons is 5 to 10-fold less efficient compared to initiation at an AUG start codon, and is decisively dependent of surrounding secondary RNA structures and sequences, notably the Kozak sequence (G/ACCAUGG) that borders the start codon (Kozak, 1981; Kozak, 1990). Further experiments revealed that FMRpolyG translation follows a classical m7G cap-dependent ribosome scanning mechanism, and that the CGG repeat expansion is located within a small upstream ORF overlapping and negatively-regulating translation of the main *FMR1* ORF, and FMRP (Kearse et al., 2016; Sellier et al., 2017; Rodriguez et al., 2020; Figure 2). Upstream ORFs (uORFs) are short open reading frames located upstream of the main protein-encoding ORF, and are thus embedded in the 5′UTR sequence of gene. Whole genome analyses revealed that most mammalian genes contain uORFs, with their majority (>75%) initiating at near-cognate codons (Ingolia et al., 2011; Lee et al., 2012; Fields et al., 2015; Johnstone et al., 2016). As a consequence of the 5′ to 3′ ribosome scanning mechanism, a ribosome translating an uORF and dissociating at its stop codon may not re-initiate to a nearby downstream ORF, even initiated by a canonical AUG start codon. Thus, presence of upstream ORFs may negatively regulate, yet not systematically, translation of downstream ORFs. However, due to the low efficiency of translation initiation at near-cognate start codons, ribosome may bypass an uORF and instead initiates at the main downstream ORF start codon, a process known as leaky scanning translation (review in Hinnebusch et al., 2016; Kearse and Wilusz, 2017; Gao et al., 2017). As uORFs act predominantly as translation negative cis-regulatory RNA elements, they are rarely expressing functional proteins and the vast majority of uORFs encode for small and instable peptides that are hardly detectable. Consistent with this, in control condition with no repeat expansions (∼30 CGG) the *FMR1* uORF encodes for a small protein of 83 amino acids (∼7 kDa), hardly detectable without inhibition of the proteasomal degradation pathway (Sellier et al., 2017). In contrast, expansions over 60–70 CGG repeats in FXTAS lengthen this uORF, resulting in expression of a protein with a polyglycine stretch, FMRpolyG, which is stable and readily detectable (Todd et al., 2013; Kearse et al., 2016; Sellier et al., 2017, Figure 2). Of interest, the FMRpolyG protein with an expansion over ∼30 glycine repeats is prone to aggregation and expression of FMRpolyG in various cell and animal models leads to the formation of cytoplasmic and intranuclear aggregates, positives for ubiquitin and p62 (Todd et al., 2013; Hukema et al., 2015; Sellier et al., 2017; Derbis et al., 2018; Hoem et al., 2019; Wenzel et al., 2019). Importantly, various mouse or rabbit antibodies independently developed and directed against FMRpolyG revealed presence of this protein within the typical eosinophilic ubiquitin-positive intranuclear inclusions in cell and tissue sections of individuals with FXTAS (Todd et al., 2013; Buijsen et al., 2014; Sellier et al., 2017; Bonapace et al., 2019; Krans et al., 2019; Dijkstra et al., 2021). Of technical interest, these antibodies are directed against the N- or C-terminal parts of FMRpolyG, but none are directly targeting the polyglycine stretch. However, recent mass-spectrometry analyses of FXTAS brain samples revealed only trace amount of FMRpolyG, casting important questions on the expression level of this protein in individuals with FXTAS (Ma et al., 2019; Holm et al., 2021). Such low levels of FMRpolyG found in proteomic analyses may originate from the high propensity of this protein to aggregates (Sellier et al., 2017), so that little FMRpolyG is present in brain extract or in fluids of individuals with FXTAS. Overall, these proteomic studies have important clinical consequences as they temper relevance of FMRpolyG as an easily quantifiable biomarker for FXTAS (Ma et al., 2019; Holm et al., 2021). Finally, expression of FMRpolyG in cell and animal models is toxic, and Drosophila or mice expressing FMRpolyG show locomotor alterations, neuronal cell loss and ultimately, premature death of these animals (Todd et al., 2013; Sellier et al., 2017). However, the mechanism of FMRpolyG toxicity is currently unclear (review in Boivin et al., 2018; Glineburg et al., 2018), with proposed alteration of the proteasome and degradation pathway (Oh et al., 2015), of the nuclear architecture and nucleocytoplasmic transport (Sellier et al., 2017), of mitochondrial functions (Gohel et al., 2019) and/or through binding to *FMR1* CGG RNA repeats (Asamitsu et al., 2021). Furthermore, it is unclear whether FMRpolyG toxicity is related to its propensity to form large cellular aggregates and/or to its localization and notably whether its import into nuclei is required for its pathogenicity. Similarly, whether FMRpolyG toxicity is mainly triggered by its polyglycine stretch, or whether bordering N- or C-terminal amino acid sequences contribute to FMRpolyG pathogenicity is currently ill-defined (Sellier et al., 2017).

In parallel to the translation initiation of a CGG repeats-containing uORF at a near-cognate start codon resulting in expression of the toxic FMRpolyG protein, RAN translation where initiation occurs directly within the CGG expansion and in all three frames was also proposed as a leading pathogenic mechanism in FXTAS (Todd et al., 2013). While modest to no translation of CGG repeats was found in the arginine frame, which is the frame of the downstream FMRP ORF, assays using highly-sensitive nanoluciferase detection methods revealed some translation of the expanded CGG repeats into the alanine frame, resulting in expression of a novel polyalanine containing protein that was named FMRpolyA (Todd et al., 2013; Oh et al., 2015; Kearse et al., 2016). However, independently developed antibodies directed against FMRpolyA failed to detect significant accumulation of this protein in cells and tissues of individuals with FXTAS, suggesting that FMRpolyA is not a major component of the intranuclear inclusions typical of this disease (Sellier et al., 2017; Krans et al., 2019). Furthermore, a mouse model expressing an expansion of CGG repeats but deleted of the *FMR1* 5′UTR sequence that contains the near-cognate start codon initiating FMRpolyG translation shows no inclusions and no locomotor or neurodegenerative phenotype. In contrast, mice expressing the same CGG expansion, but with its upstream natural *FMR1* near-cognate codon, show numerous FMRpolyG inclusions and neurodegeneration (Sellier et al., 2017). These animal models suggest that RAN translation initiating inside the CGG repeat track is not sufficiently efficient to drive inclusion formation and neuronal cell death, at least in the limited time frame of mouse longevity. Similarly, these models suggest that the sole expression of CGG repeats at the RNA level, and thus an RNA gain of function mechanism, is not sufficient to drive neuropathogenicity in these animals.

**FIGURE 3:**
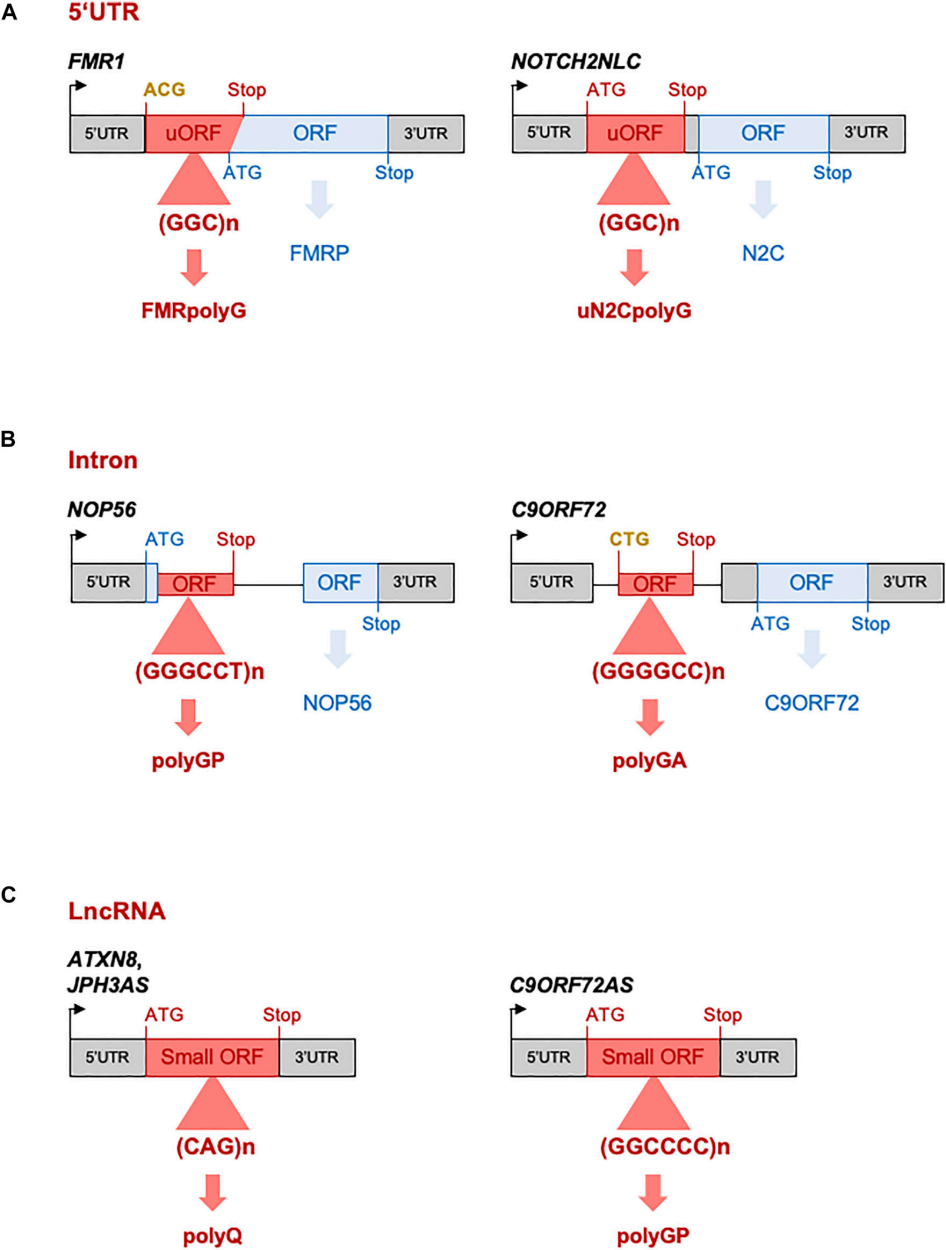
Repeat expansions located in “non-coding” regions are nonetheless translated. **(A)** CGG repeat expansions embedded in FMR1 and NOTCH2NLC 5′UTR are translated into polyglycine-containing proteins in NIID and FXTAS, respectively. **(B)** G3C2T and G4C2 repeats embedded in the first unspliced intron of the NOP56 and C9ORF72 genes are translated into poly(glycine-proline) and poly(glycine-alanine)-containing proteins in SCA36 and ALS/FTD, respectively. **(C)** CAG repeats and G2C4 repeats embedded in ATXN8, JPH3AS and C9ORF72AS long “non-coding” RNAs are translated into polyglutamine- or poly(glycine-proline)-containing proteins in SCA8, HDL2, and ALS/FTD, respectively. Initiation codons and stop codons of the ORFs containing the pathogenic expansion are indicated in red, while they are indicated in blue for the main ORFs. Near-cognate initiations codons are indicated in bold yellow.

Overall, finding that CGG repeats are translated into a novel and toxic polyglycine-containing protein clarifies the origin of the intranuclear inclusions and the pathogenic mechanism at play in FXTAS. Furthermore, these results support a novel model of pathogenicity in human diseases, where repeat expansions located in ill-defined upstream ORFs can be decoded into novel and toxic proteins through translation initiation at near-cognate start codons. Interestingly, a similar mechanism was recently identified in amyotrophic lateral sclerosis and frontotemporal dementia, where an expansion of G4C2 repeats located in the first intron of the *C9ORF72* gene is translated into a polyglycine-alanine dipeptide repeat (polyGA DPR) protein through translation initiation at a CUG near-cognate start codon located 24 nucleotides upstream of the repeats (Green et al., 2017; Tabet et al., 2018; Sonobe et al., 2018; Almeida et al., 2019; Boivin et al., 2020; Sonobe et al., 2021; Figure 3). It remains to determine whether these examples of translation initiation at near-cognate start codons are two isolated cases or reflect a more global mechanism of disease.

## Neuronal Intranuclear Inclusion Disease

Neuronal intranuclear inclusion disease (NIID, OMIM: 603472), also known as intranuclear inclusion body disease (INIBD) or neuronal intranuclear hyaline inclusion disease (NIHID), is a rare autosomal dominant genetic disorder with variable age of onset, from infant to late adult (Lindenberg et al., 1968; Michaud and Gilbert, 1981; Munoz-Garcia and Ludwin, 1986; Takahashi-Fujigasaki, 2003; Sone et al., 2016). The clinical manifestations of NIID are highly heterogenous and generally comprise variable skeletal muscle weakness associated with variable dysfunctions of the central and peripheral nervous systems, including cerebellar ataxia, parkinsonism, cognitive decline, peripheral neuropathy, and autonomic dysfunctions (Takahashi-Fujigasaki, 2003; Sone et al., 2016; Takahashi-Fujigasaki et al., 2016). However, atypical NIID clinical manifestations are increasingly described, including leukoencephalopathy, essential tremor, multiple system atrophy, retinal changes and retinopathy, amyotrophic lateral sclerosis, Alzheimer, or Parkinson disease (Okubo et al., 2019; Fang et al., 2020; Ma et al., 2020; Nakamura et al., 2020; Ng et al., 2020a; Yuan et al., 2020; Shi et al., 2021; Yang et al., 2022). Furthermore, there is increasing report of various acute symptoms of the central nervous system associated with NIID, including stroke-like episodes, migraine-like attack, epileptic seizures and/or encephalitic episodes; as well as evidences of clinical manifestations from other systems (Chen H. et al., 2020). Overall, these recent clinical observations suggest that NIID covers a much larger-than-previously-thought spectrum of clinical manifestations potentially extending to almost all systems (review in Cao Y. et al., 2021; Cao L. et al., 2021; Fan et al., 2022).

As its name imply, NIID is characterized by the presence of large eosinophilic ubiquitin-, p62-, and sumo-positive intranuclear inclusions. These aggregates are present in both neurons and glial cells in the central and peripheral nervous systems, as well as in various other tissues, including the skin, which observation can be determinant to confirm diagnosis of this multifaced syndrome (Patel et al., 1985; Kimber et al., 1998; Pountney et al., 2003; Liu et al., 2008; Sone et al., 2011; Mori et al., 2012; Chen H. et al., 2020; Zhang et al., 2021). Importantly, these intranuclear inclusions are virtually identical and indistinguishable to those observed in FXTAS (Gelpi et al., 2017; Lim et al., 2020; Toko et al., 2021). Similarly, brain MRI of individuals with NIID reveal abnormalities reminiscent of FXTAS with mild brain atrophy and white matter lesions, including T2-weighted hyperintensities in the middle cerebellar peduncle and high-intensity signals in the corticomedullary junction (Gelpi et al., 2017; Sugiyama et al., 2017; Ng et al., 2020b).

The genetic cause of NIID was recently identified as a limited expansion of ∼60 to 200–300 CGG repeats in the 5′UTR of the *NOTCH2NLC* gene (Sone et al., 2019; Ishiura et al., 2019; Deng et al., 2019; Tian et al., 2019). Of interest, an identical expansion of CGG repeats in *NOTCH2NLC* was also recently identified as the genetic cause of oculopharyngodistal myopathy type 3 (OPDM3) with variable neurological manifestations (Ogasawara et al., 2020; Yu et al., 2021). These studies highlight the variability of clinical manifestations and the ensuing complexity to diagnose these syndromes, as well as the potential overlap between OPDM and NIID. Furthermore, *NOTCH2NLC* CGG expansion is mostly reported in individuals of Asian origin, but rarely observed in European NIID cases, despite clinical similitudes and the presence of identical intranuclear inclusions (Chen Z. et al., 2020), suggesting a founder effect for this mutation, but also that NIID is genetically heterogenous with other mutations awaiting to be identified. The Notch 2 N-terminal like (*NOTCH2NL*) A, B and C genes are hominid-specific genes located on chromosome 1q21.1, which result from the duplication of the promoter region and N-terminal part (exons 1–5) of *Notch2*. As a consequence of this partial duplication, the NOTCH2NL proteins contain six epidermal growth factor (EGF)-like domains, but are deleted of the middle transmembrane and C-terminal cytoplasmic domains of Notch2. Moreover, these proteins also lack the N-terminal Notch2 peptide signal due to a modified ATG start site localization in *NOTCH2NLA* and *C*, and an intronic nucleotide variation changing *NOTCH2NLB* intron 1 splice acceptor by 8 nucleotides and thus modifying NOTCH2NLB ATG start site accordingly. Important to hominid brain size evolution, NOTCH2NL proteins regulate Notch signaling and expand human cortical progenitors. Furthermore, 1q21.1 genomic deletions or duplications potentially encompassing the *NOTCH2NLA* and B genes are associated with neurodevelopmental syndromes with microcephaly or macrocephaly, respectively, (Fiddes et al., 2018; Suzuki et al., 2018). These seminal studies highlight the importance of the NOTCH2NL proteins for neuronal progenitor proliferation and brain size expansion in human evolution, but question how a CGG repeat expansion embedded within the 5′UTR of *NOTCHNLC* can be pathogenic. First, a loss of function of the NOTCH2NLC (abbreviated N2C) protein is unlikely as the CGG repeats are located 140 nucleotides ahead of the ATG start site of this protein, and as *NOTCH2NLC* mRNA levels are found unaltered, or even increased, in blood, brain, fibroblasts and muscle samples of individuals with NIID or OPDM3 (Sone et al., 2019; Ishiura et al., 2019; Tian et al., 2019; Yu et al., 2021). Moreover, the NOTCHNLC protein is most likely expressed at very low level due to the presence of six ATG-driven upstream ORFs inhibiting its translation initiation, questioning the physiological importance of this protein (Zhong et al., 2021). Second, an RNA gain of function mechanism has been considered for NIID, with some evidences of CGG RNA accumulation in RNA foci, but with unclear pathological consequences (Yu et al., 2021; Deng et al., 2021). Third, translation of these CGG repeats into a potentially toxic protein has been recently observed in two independent reports (Boivin et al., 2021; Zhong et al., 2021; Figure 2). Importantly, these studies revealed that the *NOTCH2NLC* CGG repeats are embedded into a small upstream ORF, which is translated from a canonical ATG start site located 15 nucleotides upstream of the CGG repeats. This uORF was hence named uN2C for upstream of the NOTCH2NLC ORF. As a results of this ATG translation initiation, each GGC triplet encodes for a glycine, resulting in expression of a polyglycine-containing protein, which was named either uN2CpolyG (Boivin et al., 2021) or N2NLCpolyG (Zhong et al., 2021). Despite different names and independent identification, these two proteins are strictly identical (Figure 2). As with the majority of upstream ORFs, in control condition the N2C uORF with no expansion (∼12 CGG) encodes for a small protein (56 amino acids, ∼6 kDa), which is unstable and hardly detectable without inhibiting degradation pathways (Boivin et al., 2021). In contrast, CGG repeat expansion increases the length of this uORF, resulting in expression of a polyglycine-containing protein that is now stable and detectable. Importantly, mouse monoclonal antibodies directed against two different amino acid sequences, both located in the C-terminal part, of uN2CpolyG revealed presence of this protein in the typical ubiquitin- and p62-positive intranuclear inclusions in skin and brain sections of individuals with NIID (Boivin et al., 2021). These results were recently confirmed using an independently-developed rabbit polyclonal antibody directed against a stretch of 12 glycine followed by 11 amino acids from the uN2C sequence (Zhong et al., 2021). Furthermore, expression of this polyG protein in cell and animal models drives the formation of cytoplasmic and intranuclear inclusions, positive for ubiquitin, sumo and p62. Finally, expression of uN2CpolyG in mice is toxic, resulting in locomotor alterations, neuronal cell loss and reduced lifespan (Boivin et al., 2021). However, by which mechanism this polyG protein is toxic is unclear. Immunoprecipitation followed by mass spectrometry analyze indicate that the normal uN2C protein with a normal stretch of polyglycine (∼12 repeats) interacts with the Ku70 and Ku80 proteins, which form a scaffold for the non-homologous end joining (NHEJ) repair of DNA double-strand breaks. Interestingly, expression of uN2C (with a control number of CGG repeats) boosts the repair of DNA double strand breaks (Boivin et al., 2021). These results indicate that the small uN2C protein is potentially a novel regulator of DNA damage response, suggesting that the *NOTCH2NL* genes may encode different proteins with complementary functions for human brain development. In this model, the *NOTCH2NLB* gene, encoding the NOTCH2NLB protein, would regulate Notch signaling to promote extensive neural progenitor proliferation (Fiddes et al., 2018; Suzuki et al., 2018), but at the cost of potentially generating multiple DNA damages. In contrast, the *NOTCH2NLC* gene contains six ATG-driven upstream ORFs that reduce expression of the NOTCH2NLC protein (Zhong et al., 2021), but the first of these uORFs encodes for a small uN2C protein that may protect neuronal cells from DNA damages by stimulating the NHEJ repair mechanism. Of interest, *NOTCH2*, *NOTCH2NLA* and *B* also contains 1, 5 and 2 potential ATG-driven uORFs, respectively, but with protein sequences different from uN2C and are thus unable to interacts with the Ku70 and 80 proteins (Boivin et al., 2021; Figure 2). Note that alike *NOTCH2NLC*, *NOTCH2NLA* contains multiple ATG-driven uORFs, which may silence expression of the downstream NOTCH2NLA ORF, questioning the expression and relevance of the NOTCH2NLA and NOTCH2NLC proteins in physiological conditions.

In NIID, the polyglycine expansion alters the interaction of uN2C with the Ku proteins and reduces its DNA repair activity. However, this reduced function is likely compensated by the second NOTCH2NLC allele and is not sufficient to drive overt DNA repair alterations, as no clear evidence of DNA damage accumulation was observed in mice expressing the uN2CpolyG protein or in brain sections of NIID individuals (Boivin et al., 2021). Interestingly, expression of this polyG protein promotes alterations of the nucleocytoplasmic transport (Zhong et al., 2021), which is reminiscent of the pathogenicity described for FMRpolyG in FXTAS (Sellier et al., 2017), as well as for DPR proteins expressed from the G4C2 repeats in the C9ORF72 gene in ALS/FTD (Jovičić et al., 2015; Hayes et al., 2020). However, it remains to investigate by what molecular mechanisms the uN2CpolyG and FMRpolyG proteins may dysregulate nucleocytoplasmic traffic. It also remains to determine whether RAN translation of the NOTCH2NLC repeats, with initiation within the CGG expansion and protein expression in all three frames, may occur and contribute to the pathogenicity in NIID.

Overall, these studies suggest the existence of a novel class of human genetic disorder, the polyG diseases, where expanded CGG repeats are embedded in small upstream ORFs and consequently, are translated into novel polyglycine-containing proteins that are toxic and forms ubiquitin-positive inclusions (Todd et al., 2013; Sellier et al., 2017; Boivin et al., 2021; Zhong et al., 2021). It remains to determine whether this group of disease is limited to NIID and FXTAS, or whether it may include other pathologies with related symptoms and/or similar histopathological features. Candidate pathologies may include mutations, yet to be identified, in NIID cases negative for the NOTCH2NLC mutation, but that nevertheless present the typical clinical and histopathological features of that syndrome (Chen Z. et al., 2020). Similarly, it remains to explore whether translation of expanded CGG repeats, yet to be identified, may underlie the presence of intranuclear inclusions of unknown origins (review in JM Woulfe, 2007), notably Marinesco bodies, which are eosinophilic ubiquitin-positive nuclear inclusions found in pigmented neurons of the substantia nigra and locus ceruleus in aged human brain (Yuen and Baxter 1963; Odagiri et al., 2012). Finally, the recent identification of CGG repeat expansions in the OPDM and OPML neuromuscular disorders, which are characterized by the presence of eosinophilic ubiquitin-positive intranuclear inclusions, may potentially represent novel examples of polyG diseases.

## Oculopharyngodistal Myopathies

Oculopharyngodistal myopathy (OPDM, OMIM: 164310) is a rare adult-onset and slowly progressive autosomal dominant disease characterized by ptosis, external ophthalmoplegia, dysphagia and dysarthria, associated to facial and distal limb muscle weakness (Satoyoshi and Kinoshita 1977; Uyama et al., 1998; Minami et al., 2001; van der Sluijs et al., 2004; Lu et al., 2008; Durmus et al., 2011; Zhao et al., 2015). Serum creatine kinase levels are usually normal or mildly increased in individuals with OPDM, and skeletal muscles shows moderate fibrosis and small angular fibers. Besides these non-specific myopathic changes, OPDM histopathology is characterized by the presence of cytoplasmic rimmed vacuoles (RVs) and typical eosinophilic nuclear inclusions (NIs), which are both p62-and ubiquitin-positive (Zhao et al., 2015; Deng et al., 2020; Saito et al., 2020; Kumutpongpanich et al., 2021; Matsubara et al., 2021; Xi et al., 2021). Of interest, these intranuclear inclusions are also observed in skin sections of individuals with OPDM cases (Ogasawara et al., 2021), and are reminiscent of the typical inclusions observed in FXTAS and NIID. Importantly, the mutations causing OPDM were recently identified as similar expansions of ∼70 to 200–300 CGG repeats located within the 5′UTR of two different genes, LRP12 and GIPC1 (Ishiura et al., 2019; Deng et al., 2020; Kumutpongpanich et al., 2021; Xi et al., 2021). Furthermore, an expansion of CGG repeats in the 5′UTR of the NOTCH2NLC gene was identified in OPDM cases with variable neurological manifestations (Ogasawara et al., 2020; Yu et al., 2021), and an expansion of CGG repeats within the 5′UTR of the RILPL1 gene was recently reported in medRxiv in affected individuals of a large Chinese family with OPDM (Yang et al., 2021). Consequently, OPDM is now distinguished by its genetic cause with oculopharyngodistal myopathy type 1 (OPDM1) caused by a CGG repeat expansion in LRP12, OPDM type 2 is caused by a CGG expansion in GIPC1, OPDM3/NIID1 is caused by a CGG expansion in NOTCH2NLC and OPDM4 is potentially associated with a CGG expansion in RILPL1. There is probably a founder effect for these mutations as the CGG expansion in LRP12 is more prevalent in Japan compared to China, while an inverse distribution is observed for the CGG expansion in GIPC1.

The mechanism of toxicity in OPDM is yet to be identified but a loss of function is unlikely as the CGG repeat expansions are located ahead of the ATG start sites of the GIPC1, LRP12, NOTCH2NLC, and RILPL1 proteins. Furthermore, expression of the GIPC1 protein is unaltered in tissue samples from individuals with OPDM2 (Deng et al., 2020). A mechanism of CGG RNA gain of function and/or of translation into pathogenic proteins remains to be explored, but it is noteworthy that oculopharyngeal muscular dystrophy, a neuromuscular disease with clinical manifestations related to OPDM, is also caused by a GC-rich repeat expansion. Oculopharyngeal muscular dystrophy (OPMD, OMIM: 164300) is a late-onset and slowly progressive autosomal dominant neuromuscular disease characterized by proximal muscle weakness, ptosis, and swallowing difficulty. OPMD is caused by an extension of 1–8 repeats of a GCG trinucleotide stretch located in PABPN1 gene, encoding the polyadenylate-binding protein 2 (PABP2). The N-terminal part of this protein contains a short polyalanine tract (Met-(Ala)10x-Gly-(Ala)2x … ), and in OPMD a small GCG expansion adding 1 to 8 alanine, or a missense mutation of the +12 glycine into an alanine resulting in a contiguous stretch of 13 alanine, leads to a modified PABP2 protein that interfere with the cellular traffic of polyadenylated RNA (Brais et al., 1998; Robinson et al., 2006). This is reminiscent of congenital polyalanine diseases, which are caused by small GCN repeat expansions encoding polyalanine tracts located within eight different transcription factors (Table 1). These elongated polyalanine stretches of relatively small size (<35 repeats) are proposed to cause loss of function and/or dominant negative effect on the transcriptional activities of these factors, ultimately leading to developmental disorders (review in Albrecht and Mundlos, 2005; Shoubridge and Gecz, 2012). Of interest, similar congenital aetiologies are observed in individuals with deletion and/or loss of function mutations in the genes coding for these transcription factors, strengthening the hypothesis that these short polyalanine tracts are pathogenic through a loss of function mechanism. In contrast, the CGG repeat expansions in OPDM1 to 4 are longer and localized in the 5′UTR, and not in the coding sequences of the GIPC1, LRP12, NOTCH2NLC, and RILPL1 proteins. Thus, expansions of ∼70 to 200–300 CGG repeats in OPDM are likely to be pathogenic by a different mechanism than the small (11–18) polyalanine tract that alters PABP2 protein functions in OPMD.

## Oculopharyngodistal Myopathy With Leukoencephalopathy: The Missing Piece of a Disease Continuum?

Overall, it is striking to note that the OPDM, FXTAS, and NIID diseases share overlapping clinical manifestations and identical histopathological features, with the presence of characteristic eosinophilic ubiquitin-positive nuclear inclusions (NIs). Furthermore, these diseases are caused by similar genetic causes, namely microsatellite expansions of identical sequence and size (60–70 to 200–300 CGG repeats). These data suggest that these disorders may belong to a continuum of neuromuscular and neurodegenerative diseases (Figure 1). Importantly, this hypothesis is reinforced by the recent discovery that both NIID and OPDM3 can be caused by an identical CGG repeat expansion in NOTCH2NLC (Sone et al., 2019; Ishiura et al., 2019; Deng et al., 2019; Ogasawara et al., 2020; Yu et al., 2021), as well as by the identification of a novel and rare clinical entity with clinical features of both NIID and OPDM, and that was consequently named oculopharyngodistal myopathy with leukoencephalopathy (OPML, OMIM: 618637) (Ishiura et al., 2019). OPML is characterized by muscle dystrophy and weakness, especially of the ocular, pharyngeal, facial, and distal limb muscles, associated with leukoencephalopathy, brain atrophy, and T2 hyperintensity signals in the white matter and sign of tremor and ataxia in some individuals. Interestingly, OPML is caused by an expansion of CGG repeats located within the ill-defined LOC642361/NUTM2B-AS1 genetic region, which is transcribed but predicted as non-coding (Ishiura et al., 2019). Thus OPML, alike FXTAS, OPDM, and NIID, is caused by a CGG repeat expansion embedded in a genetic region predicted as non-coding, questioning the pathogenic mechanisms underlying these diseases. In that aspect, it was recently unveiled that CGG repeat expansions located in the 5′-untranslated regions of the FMR1 or NOTCH2NLC genes are nonetheless translated into toxic polyglycine-containing proteins through canonical translation initiation at canonical AUG, or near-cognate, start codons located upstream of the repeats (Todd et al., 2013; Sellier et al., 2017; Boivin et al., 2021; Zhong et al., 2021; Figure 3). Similarly, G4C2 and G3C2T repeat expansions are translated into toxic poly(glycine-alanine)- and poly(glycine-proline)-containing proteins in ALS/FTD and SCA36, despite these repeats being localized in the first intron of the C9ORF72 and NOP56 genes, respectively. These intronic regions are unspliced in patients and, alike in FXTAS and NIID, repeat expansions are translated through initiation at canonical AUG, or near-cognate, start codons located upstream of the repeats (Green et al., 2017; Tabet et al., 2018; Sonobe et al., 2018; Almeida et al., 2019; Boivin et al., 2020; McEachin et al., 2020; Sonobe et al., 2021; Figure 3). These results are reminiscent of pioneering studies showing that CAG expanded repeats located within the ATXN8 and JPH3AS RNAs, which were initially considered as long non-coding RNAs but were later on found to be carrying small ORFs, are translated through canonical ATG-initiated translation into toxic polyglutamine proteins in SCA8 and HDL2 (Moseley et al., 2006; Wilburn et al., 2011; Figure 3). Finally, it is consistently reported that various repeat expansions can be RAN translated into toxic proteins through initiation directly within the repeats and in all three frames (Zu et al., 2011; review in; Cleary et al., 2018; Nguyen et al., 2019). Thus, whether the CGG repeat expansions causing OPML and OPDM pathologies, which are located in genetic sequences predicted as non-coding, are nonetheless translated into potentially toxic proteins accumulating into ubiquitin-positive inclusions, is an exciting question that remains to be addressed.

## Expansion Without Anticipation in NIID and OPDM?

In contrast to familial anticipation and correlation between expansion lengths and disease severity and/or age of onset observed in various microsatellite diseases (FXS, polyQ disorders, and myotonic dystrophy type 1, etc.), there are only few examples of anticipation reported in NIID and OPDM families, and a limited correlation between the size of CGG expansions and the age of onset in OPDM1 and OPDM2, with a *r* ^2^ of 0.188 and 0.158, respectively, (Kumutpongpanich et al., 2021; Xi et al., 2021). On the contrary, there are increasing reports of individuals carrying large CGG expansions (>200–300 repeats) in the *NOTCH2NLC*, *GIPC1,* and *LRP12* genes and who are free of overt clinical manifestations (Deng et al., 2020; Ogasawara et al., 2020; Deng et al., 2021; Fukuda et al., 2021; Kumutpongpanich et al., 2021; Yu et al., 2021). Molecular analyses indicate that these individuals present DNA hypermethylation associated to promoter silencing and loss of expression of the allele carrying these large CGG expansions. These results have important consequences. First, they indicate that a partial reduction in expression and/or haploinsufficiency of the *NOTCH2NLC*, *LRP12,* and *GIPC1* genes are not associated with overt clinical manifestations, dismissing the hypothesis of a loss of function mechanism in OPDM and NIID. Second, these data also provide some molecular bases to the complex pedigree observed in these diseases, with increasing reports of asymptomatic ascendants who nevertheless carry the causative mutation, but of longer size (over 200–300 CGG repeats) compared to their affected descendants who typically carry an allele with 60 to 200–300 CGG repeats. Interestingly, of the ∼10 currently reported examples of asymptomatic individuals with large expansions and who have transmitted shorter repeats to their NIID or OPDM-affected descendants, all were father to infant transmission cases, suggesting a bias of expansions contraction in male germinal cells (Deng et al., 2020; Deng et al., 2021; Fukuda et al., 2021; Kumutpongpanich et al., 2021; Yu et al., 2021). Of interest, a similar bias of transmission was observed in carriers of the *FMR1* CGG repeat premutation where contraction occurred more often in paternal than in maternal transmission (Nolin et al., 2019). Moreover, it is noteworthy that a difference in parental gender transmission is observed in other microsatellite diseases, such as myotonic dystrophy type 1 where large expansions of CTG repeats occurs mainly through maternal transmission (review in Lanni and Pearson, 2019), while in Huntington disease, maternal transmission mainly results in CAG repeat contraction, whereas paternal transmission is associated with CAG repeat expansion (Aziz et al., 2011).

**FIGURE 4:**
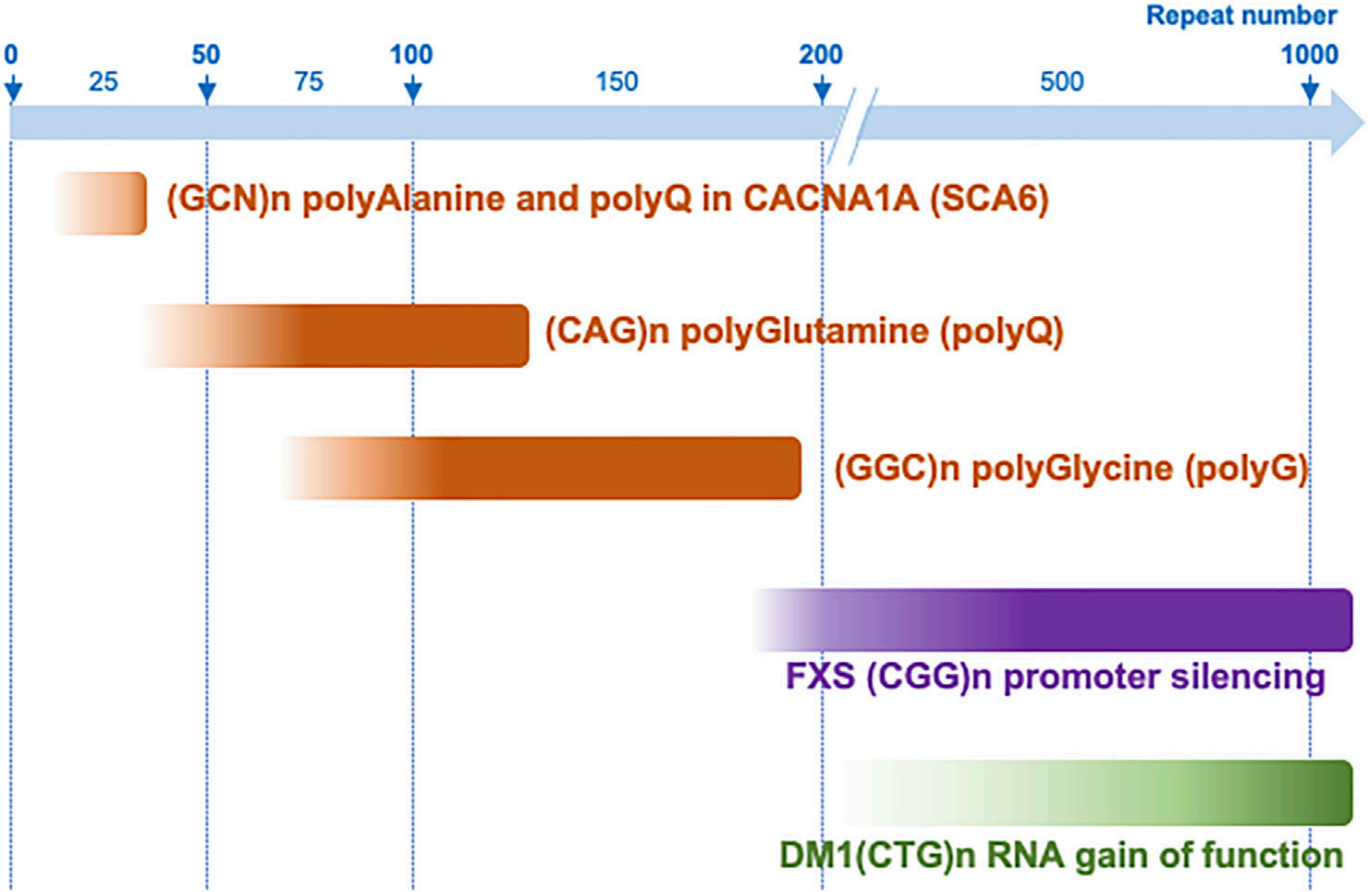
Repeat expansion range of toxicity in different microsatellite diseases. CGG repeat expansions are pathogenic between ∼60–70 and 200–300 repeats when they are expressed into toxic polyglycine-containing proteins, but over 200 repeats when they promote DNA epigenetic changes, promoter silencing and a loss of function mechanism, such as in FXS. This range of protein toxicity is to be compared to other translated microsatellite diseases such as polyalanine-containing proteins that are generally toxic with 10–35 GCN repeats, while polyQ proteins are generally pathogenic with longer CAG expansions (∼40–80–200 repeats), with the exception of short polyglutamine stretch altering functions of the calcium voltage-gated channel subunit alpha1A (CACNA1A) in SCA6. In contrast, an RNA gain of function mechanism such as titration of the MBNL RNA binding proteins in DM1, require much longer repeat expansions.

Overall, these studies suggest that in NIID and OPDM, the causing CGG expansions are pathogenic in a narrow range of size, with a lower limit of 60–70 repeats, below which they are likely not long enough to be expressed in stable and toxic proteins, and a “protective” threshold around 200 to 300 CGG repeats, over which carriers of large expansions benefit from the transcriptional silencing of the toxic allele (Figure 4). However, these intriguing observations are currently limited to a small number of clinical cases, and larger studies will be required to refine the correlation between CGG expansion sizes and age of onset and/or clinical severity in these disorders.

## Putative Mechanisms of polyG Proteins Toxicity

FXTAS and NIID are characterized by the translation of CGG repeats into pathogenic FMRpolyG and uN2CpolyG proteins, respectively. These two proteins form similar ubiquitin-positive inclusions, are toxic in cell and animal models and possess an identical polyglycine stretch, suggesting a common mechanism of toxicity. However, this raises numerous questions, notably it is unclear whether these polyG proteins are toxic under their aggregated or soluble forms. Similarly, it remains to explore whether their cellular localization, notably their import into the nucleus, is required for their pathogenicity. In that aspect, overexpression of FMRpolyG or uN2CpolyG in transformed cell lines mainly results in their accumulation in cytoplasmic aggregates, while these proteins are mainly found in intranuclear inclusions in patient tissues. Thus, it remains to clarify the mechanism responsible of polyglycine-containing proteins nuclear import in these diseases. Furthermore, FXTAS and NIID are neurodegenerative diseases, but *FMR1* and *NOTCHNLC* expression are not restricted to neurons, and intranuclear inclusions are widely observed outside of the CNS in individuals with FXTAS and NIID (Greco et al., 2006; Hunsaker et al., 2011; Yamaguchi et al., 2018; Chen H. et al., 2020). However, presences of these inclusions are associated with only limited tissue dysfunctions and/or sub-clinical observations. In consequence, the mechanisms underlying the cellular specificity of FMRpolyG or uN2polyG toxicity remain to be determined. Finally, whether the pathogenicity of these proteins originates uniquely from their polyglycine stretches, or whether there is a toxic contribution of their bordering N- or C-terminal amino acid sequences is ill-defined. In that aspect, expression of a polyglycine protein deleted of any *FMR1* or *NOTCH2NLC* bordering sequence is toxic in cell models, however not as much as the full-length FMRpolyG or uN2polyG proteins (Sellier et al., 2017; Boivin et al., 2021). Furthermore, C-terminal tagging of the FMRpolyG protein by the cherry fluorescent protein prevents its aggregation, while fusion of FMRpolyG with the GFP promotes its stability and increases its expression (Sellier et al., 2017; Derbis et al., 2018). These data suggest that the sequences bordering the polyglycine stretch of FMRpolyG or uN2polyG may contribute to their pathogenicity. However, this remains to be confirmed, notably in animal models.

## Conclusion

Translation of CGG repeat expansions into simar polyglycine-containing (polyG) proteins in two neurodegenerative diseases with overlapping clinical manifestations and nearly identical histopathological features, FXTAS and NIID, suggest the existence of a novel class of human genetic disorders, the polyG diseases. This model is inspired by the translation into toxic polyalanine- or polyglutamine-containing proteins of GCN or CAG repeat expansions embedded in the ORFs of diverse genes, resulting in the polyAla or polyQ diseases, respectively. Furthermore, these data confirm that expanded repeats localized in human genomic regions predicted as “non-coding” can nevertheless be translated into novel pathogenic proteins, notably through classical initiation at canonical AUG, or near-cognate, start codons. In that aspect, the similarities of clinical manifestations and histopathological characteristic between FXTAS, NIID, OPDM, and OPML question whether CGG repeats also located in sequences predicted as non-coding, such as the *LOC642361/NUTM2B-AS1* locus or the 5′UTR of the *LRP12* and *GIPC1* genes, are similarly translated into novel and potentially pathogenic polyG proteins. Importantly, a similar mechanism of toxicity for NIID, FXTAS and potentially other diseases, suggests that therapeutic strategies targeting the common CGG repeats/polyglycine-stretch could be of interest for the whole group of polyG diseases (review in Xu et al., 2021). In that aspect, constant efforts during the last decade to develop small pharmacological molecules specifically binding CGG RNA repeats *in vitro*, and that now efficiently decrease expression of the toxic FMRpolyG in cell and animal models provide exciting therapeutic hope for these disorders (Disney et al., 2012; Qurashi et al., 2012; Tran et al., 2014; Yang et al., 2016; Green et al., 2019; Verma et al., 2020; Asamitsu et al., 2021; Haify et al., 2021; Malik et al., 2021b; Konieczny et al., 2021). In parallel, major advances in antisense oligonucleotides (ASO) chemistry to reduce their toxicity and improve their biodistribution and cellular uptake open new hope to identify ASOs sequences targeting these CGG repeats to promote their RNA degradation and/or block their translation into toxic polyglycine-containing proteins, as recently reported in animal models of FXTAS (Rodriguez et al., 2020; Derbis et al., 2021).
